# Supplementary material for: Assessment of COVID-19 vaccine acceptance and its associated factors in Debre Berhan City, Ethiopia, 2022
Source: PLoS One. 2023 Nov 16;18(11):e0288321. doi: 10.1371/journal.pone.0288321 (PMC10653532; doi:10.1371/journal.pone.0288321)
Supplement: S1 File — (DOCX) [file pone.0288321.s001.docx]

**Part 1: Informed consent**

Code number ----------------------------------

I understand that the purpose of this study is to assess COVID-19 vaccine acceptance and its associated factors in Debre Berhan City, and I am also aware that participation in this study is entirely voluntary, with no monetary or other incentives. In addition, the information that is given is kept confidential and is not exposed to another third party.

This questionnaire will be filled out only if you agree to take part in the study, and I sincerely ask you to give your genuine and true responses to the questions provided.

So, would you agree to participate in a study?

Yes ---------------

No---------------

Signature of participant _________________

Name and Signature of the data collector who sought the consent______________

Name and signature of the supervisor ____________________ Date_________**Part I: Socio-demographic characteristics of the participant**

| **S.N** | **Questions** | **Response category** |
| --- | --- | --- |
| 101 | How old are you? | Age in year______________ |
| 102 | Sex | 1. Male 2. Female |
| 103 | What is your Marital status? | 1. Single 2. Married 3. Separated 4. Widowed 5. Divorced |
| 104 | What is your Educational Status? | 1. Unable to read and write 2. Primary education 3. Secondary education 4. Tertiary and above |
| 105 | What is your Ethnicity? | 1. Amhara 2. Oromo 3. Tigre 4. Other(specify)____________ |
| 106 | What is your religion? | 1. Orthodox 2. Protestant 3. Muslim 4. Catholic 5. Other (specify) ______ |
| 107 | What is your occupational status? | 1. Unemployed 2. Civil servant 3. Merchant 4. NGO employee 5. Day laborer 6. Student 7. Other(specify)________________ |
| 108 | What is your average monthly income in Birr? | ______________ birr |

**Part II. Acceptance of COVID-19**

| S.N | Item | Responses |
| --- | --- | --- |
| 201 | Have you vaccinated for COVID-19 vaccine? | 1. Yes 2. No |
| 202 | If your answer is “No” for question number 201, Reasons for Not Taking COVID-19 Vaccine? Multiple option is allowed | 1. Inadequate data about the safety of the vaccine 2. Fear of adverse effects of the vaccine 3. A concern on the vaccine being ineffective 4. Vaccine causing COVID-19 5. I prefer other ways of protection 6. Prior adverse reaction to any vaccine 7. Unreliable, due to short time for development |
| 203 | If “NO’’ for Q 201 will you get vaccinated if you get COVID-19 vaccine? | 1. Yes 2. No |

**Part III: Health related factors and COVID-19 experience**

| **S/N** | **Items** | **Responses** |
| --- | --- | --- |

| 301 | Do you have history of chronic illness? | 1. Yes 2. No |  |  |
| --- | --- | --- | --- | --- |
| 302 | If your answer is “Yes” for question number 301, what is your illness? | 1. Hypertension 2. Diabetes 3. Heart disease 4. Stroke 5. HIV/AIDS 6. Abdominal pain 7. Mental illness 8. Other (specify)_____ | | |
| 303 | Have you been previously infected with COVID-19? | 1. Yes | 1. No |  |
| 304 | Have you ever contacted someone with a COVID-19 patient? | 1. Yes | 1. No |  |
| 305 | Have you laboratory test for COVID-19 disease? | 1. Yes | 1. No |  |
| 306 | If your answer is “Yes” for question number 205, what was the result? | 1. Negative 2. Positive | |  |

**Part IV: Knowledge and Attitude Factors**

COVID-19 related knowledge questions

| **S/N** | **Items** | **Responses** |
| --- | --- | --- |
| 401 | Do you know about the COVID-19 vaccine? | 1. Yes 2. No |
| 402 | Do you know about the effectiveness of the COVID- 19 vaccine? | 1. Yes 2. No |
| 403 | Is it dangerous to use an overdose of COVID-19 vaccines? | 1. Yes 2. No |
| 404 | Does COVID-19 vaccination increase allergic reactions? | 1. Yes 2. No 3. I don’t know |
| 405 | Does taking COVID-19 increase your risk of other diseases? | 1. Yes 2. No 3. I don’t know |

**Part V: Attitude towards COVID-19 vaccine**

| **S/N** | **Items** | **Responses** |
| --- | --- | --- |
| 501 | Is newly discovered COVID-19 vaccine is safe? | 1. Agree 2. Disagree |
| 502 | COVID-19 vaccine is essential for us? | 1. Agree 2. Disagree 3. Undecided |
| 503 | COVID vaccine developed in Europe and America are safer than those made in other world countries? | 1. Agree 2. Disagree |
| 504 | May you encourage your family/friends/relatives to get vaccinated? | 1. Agree 2. Disagree 3. Undecided |
| 505 | COVID-19 vaccine can prevent covid-19 infection? | 1. Agree 2. Disagree |
| 506 | It is not possible to reduce the incidence of COVID-19 without vaccination? | 1. Agree 2. Disagree 3. Undecided |
| 507 | The COVID-19 vaccine should be distributed fairly to all of us? | \| 1. Agree 2. Disagree \| \|  \| \|  \|  \| \| --- \| --- \| --- \| --- \| --- \| --- \| \|  \|  \|  \| |

**Part VI: Vaccine related factors**

| S.N | Item | Responses |
| --- | --- | --- |
| 601 | Have you received all the necessary vaccination in your lifetime? | 1. Yes 2. No |
| 602 | Do you have Trust in the health system regarding to the vaccine? | 1. Yes 2. No |
| 603 | COVID-19 preventive measures?  Multiple option is allowed | 1. Eating a balanced diet  2. Social distancing  3. Exercising regularly  4. Covid-19 vaccine  5. Hand washing and  6. face mask  7. Other (specify) ______ |
| 604 | What is the Source of information regarding to covid-19 vaccine?  Multiple option is allowed | 1. Television 2. Newspapers/Magazines 3. Websites / social media 4. Friends/Family 5. Other (specify) ______ |

**Qualitative tool**

**Data collector guide:**

| 1. What is your opinion regarding the COVID-19 vaccine? 2. What are your beliefs regarding the COVID-19 vaccine used in the massive vaccination drive (by the government) to eliminate COVID-19 infection? 3. What are the key points that you hesitate to take COVID-19 vaccine? 4. What are the key factors/ motivators that drive you to hesitate to COVID-19 vaccine? |
| --- |

Thank you!
