# Supplementary material for: Assessment of COVID-19 vaccine acceptance and its associated factors in Debre Berhan City, Ethiopia, 2022
Source: PLoS One. 2023 Nov 16;18(11):e0288321. doi: 10.1371/journal.pone.0288321 (PMC10653532; doi:10.1371/journal.pone.0288321)
Supplement: Attachment — Submitted filename: plose one comment.docx [file pone.0288321.s003.docx]

Title; Assessment of COVID-19 Vaccine Acceptance and its associated factors in Debre Berhan City, Ethiopia, 202

The title ; the title of this study is timely and good.

Abstract; the authors should summarize the challenge of COVID-19 and mention why they needed to conduct this study,

The objective of the study should be mentioned.

Better to summarize the methods in short and precise way. Better to use general terms to conclude the main findings of the research.

INTRODUCTION; the introduction parts lacks basic information regarding COVID 19 vaccine acceptance and its challenge, literature review, the burdens of the problems and the expected outcome should also be well stated,…

What is the need of putting specific objectives?

The authors should explain about the study area…..

In the inclusion criteria, why the authors need to include individuals who lived > 6months?

The sample size determination procedure is not clear……how the study subjects were selected?

Figure 1: Schematic presentation of sampling procedure, where is figure 1?

In the result part better to focus on the main findings rather than narrating the results.

The discussion part should focus on the findings and the factors associated with COVID 19 vaccin acceptances.

In general the manuscript needs grammar, coherence and spelling corrections.
